# Internal security vs. external options: a goal-shielding explanation of how employability differentially shapes unethical pro-organizational behavior

**DOI:** 10.3389/fpsyg.2025.1605697

**Published:** 2025-09-17

**Authors:** Yulin Niu, Dandan Liu, Xiongying Niu

**Affiliations:** International Business School, University of International Business and Economics, Beijing, China

**Keywords:** employability, unethical pro-organizational behavior, bottom-line mentality, risk propensity, goal-shielding theory

## Abstract

**Introduction:**

The role of employability in employees' unethical decision-making remains unclear. Drawing on goal shielding theory, this research investigates how internal and external employability differentially influence unethical pro-organizational behavior (UPB). We propose that these relationships are mediated by bottom-line mentality (BLM) and moderated by employees' risk propensity.

**Methods:**

We conducted two multi-wave survey studies in China. Study 1 (*N* = 273) provided an initial test of the direct and curvilinear relationships. Study 2 (*N* = 316) tested the full mediated moderation model, with data on employability and risk propensity collected at Time 1, BLM at Time 2, and UPB at Time 3.

**Results:**

The findings revealed distinct pathways for the two dimensions of employability. Internal employability was negatively associated with UPB, an indirect effect mediated by a reduction in BLM. This negative indirect relationship was significantly stronger for employees with low risk propensity. Conversely, we found an indirect, inverted *U*-shaped relationship between external employability and UPB via BLM, such that moderate external employability was associated with the highest levels of BLM and UPB. This non-linear effect was not moderated by risk propensity.

**Discussion:**

These findings offer a new theoretical lens for understanding the motivational mechanisms behind UPB, highlighting how internal job security can reduce unethical conduct while moderate external opportunities may paradoxically increase it. The results provide practical implications for organizations on managing employee career development to prevent unethical behavior.

## 1 Introduction

In today's dynamic business environment, organizations need employees with up-to-date knowledge and skills (Lazarova and Taylor, 2009), which means lifelong employment can no longer be guaranteed (Nelissen et al., 2017). As a result, employability has become an increasingly important aspect of career development (De Vos et al., 2021). This indicates that employees need to concern themselves not only with career prospects within their current organization but also with their potential for finding employment in the broader labor market (Rothwell and Arnold, 2007). Given the career uncertainty introduced by this emphasis on employability, its influence on employees' ethical decision-making warrants in-depth exploration. Existing studies indicate that individuals facing threats or uncertainty are more prone to unethical conduct (Mishra et al., 2022). This principle is robust across diverse settings, from traditional workplaces to emerging digital ecosystems where factors like intense financial pressure and market anonymity create fertile ground for fraud (Upadhyay and Upadhyay, 2025). Such exploration is therefore particularly important in understanding behaviors such as unethical pro-organizational behavior (UPB), which refers to work behaviors conducted with the intention (at least partially) to benefit the organization, but which violate core societal values, norms, laws, or standards of proper conduct (Umphress et al., 2010).

Despite the importance of this issue, researchers have rarely explored the relationship between employability and UPB. Most existing studies on individual employability have focused on consequences like career transitions (De Vos et al., 2021; Farashah et al., 2023) and employee retention (Acikgoz et al., 2016; Nelissen et al., 2017), while its implications for risky ethical behaviors have been largely overlooked. Similarly, scholars have examined antecedents of UPB from various perspectives (Mishra et al., 2022), including personality traits (Castille et al., 2018), organizational culture (Vem et al., 2023), and leadership styles (Bryant and Merritt, 2021). However, there has been a noticeable lack of attention to career-related factors, particularly employability. This oversight limits our understanding of the full spectrum of influences that drive UPB, leaving a critical gap in the literature.

This research gap is particularly problematic given the inherent complexity of how employability might influence ethical decision-making. On one hand, some employees might engage in UPB to demonstrate their value to their current employer, especially when they perceive limited external opportunities. On the other hand, employees with strong external employment prospects might avoid UPB for fear of damaging their broader career reputation. This apparent contradiction suggests that employability encompasses multiple dimensions, each with potentially unique impacts on ethical behavior in organizations. According to Rothwell and Arnold's (2007) conceptualization, internal employability refers to a worker's ability to remain employed with their current employer, while external employability denotes the ability and willingness to move to a similar or another job in another company. Though correlated, these dimensions capture distinct aspects of employability that may have differential effects on employees' ethical decision-making processes (Guilbert et al., 2016; Spurk et al., 2016).

To understand these complex relationships, we draw on goal-shielding theory, which provides a robust theoretical framework for examining how employability influences UPB. Goal-shielding theory suggests that when individuals focus on achieving a primary goal, they automatically inhibit alternative goals that might interfere with attaining the focal goal (Shah et al., 2002). Through this cognitive process, individuals become more likely to engage in behaviors that facilitate goal attainment and avoid those that do not, sometimes at the expense of ethical considerations (Kruglanski et al., 2018). Greenbaum et al. (2020) further conceptualized bottom-line mentality (BLM) as a manifestation of goal shielding, where there is a sole focus on the bottom line, ignoring other considerations including ethical ones. This theoretical lens helps explain why different dimensions of employability might have distinct impacts on employees' propensity to engage in UPB.

For employees with low internal employability, job insecurity within their current organization becomes a pressing concern (Cuyper et al., 2008). This insecurity may trigger a goal-shielding process where securing their position becomes the primary goal, potentially leading to a bottom-line mentality focused solely on performance outcomes. As a result, these employees might disregard ethical considerations that could interfere with achieving recognition or rewards necessary for maintaining their position. In contrast, high internal employability may reduce this pressure, allowing employees to consider a broader range of goals, including adherence to ethical standards (Mawritz et al., 2024).

The relationship between external employability and UPB, however, may be more complex. Unlike low internal employability, a lack of external employability does not necessarily threaten current employment (Rothwell and Arnold, 2007). When employees recognize limited opportunities elsewhere, they may align their long-term individual interests more closely with their current organization's success, including its ethical reputation. At the other end of the spectrum, employees with high external employability possess transferable skills and knowledge that provide numerous external alternatives (Guilbert et al., 2016). This career security may reduce incentives to take ethical risks that could harm their reputation in the broader labor market. Most interestingly, employees with moderate levels of external employability—who find themselves in a “good but not enough” situation—may experience heightened uncertainty about their career prospects. This uncertainty could lead them to focus narrowly on improving performance to prove their value, potentially at the expense of ethical considerations (Freitas et al., 2002; Webster and Kruglanski, 1994).

Given that UPB inherently involves risk-taking (Umphress et al., 2010), and individuals vary considerably in their tendency to take risks (Meertens and Lion, 2008; Zhang et al., 2019), it is also important to consider how employees' risk propensity might moderate the relationships between employability and UPB. Risk propensity refers to an individual's general tendency to take or avoid risks across different domains (Nicholson et al., 2005). In the workplace context, employees with higher risk propensity may exhibit more resilience toward external threats, such as job loss (Qu et al., 2025). These employees are generally more comfortable with uncertainty (Sitkin and Pablo, 1992), whereas those with low risk propensity tend to avoid risks and prioritize stability and security (Qu et al., 2025). Consequently, risk propensity may influence the extent to which employability triggers goal-shielding processes that lead to bottom-line mentality and ultimately to UPB.

**Figure 1:**
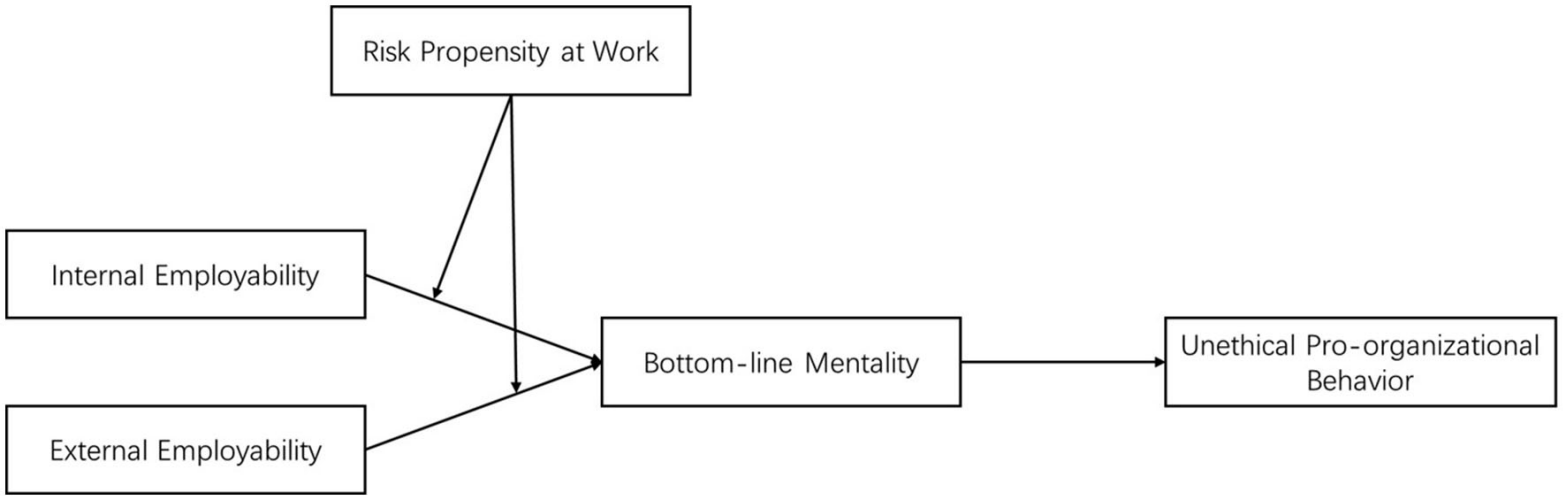
Theoretical model.

In response to these research gaps, this paper develops and tests a theoretical model to untangle the complex effects of employability. Drawing the preceding threads together, our model (see Figure 1) posits that internal and external employability differentially influence UPB through the mediating mechanism of bottom-line mentality (BLM). We further propose that an employee's risk propensity acts as a critical boundary condition, moderating the indirect effect of internal and external employability.

We empirically validate this model through a multi-wave, two-study design in China. Study 1 provides an initial test of the direct and curvilinear relationships between the two forms of employability and UPB. Study 2 then replicates these findings and examines the full mediated moderation model. By doing so, this research contributes to the literature in three primary ways: first, by examining the impacts of internal employability and external employability on UPB, we identify a new antecedent of UPB and provide insights into bridging the career and UPB literature, which has been previously underexplored. Second, by investigating the mediating role of BLM, our research establishes a theoretical basis for understanding why the two forms of employability influence UPB differently. Third, by testing the moderating role of risk propensity, we identify unique boundary conditions under which BLM transmits the influence of employability on UPB.

## 2 Theoretical development and hypotheses

### 2.1 Goal-shielding theory

Goal-shielding theory provides a robust framework for understanding how individuals prioritize and pursue their goals, particularly when faced with competing objectives. According to this theory, when individuals strongly commit to a primary goal, they automatically inhibit accessibility to alternative goals that might compete for limited cognitive resources or interfere with the attainment of the focal goal (Shah et al., 2002). This cognitive mechanism operates largely automatically rather than through conscious deliberation, allowing individuals to maintain focus and persistence in goal pursuit despite potential distractions or competing priorities.

Central to goal-shielding theory is the concept that this inhibition process is influenced by several key factors. First, the strength of commitment to the focal goal significantly determines the intensity of the shielding effect; stronger commitment leads to more powerful inhibition of alternative goals (Shah et al., 2002). Second, the type of commitment matters—whether it is driven by aspirations and ideals (“promotion-focused”) or obligations and responsibilities (“prevention-focused”) affects how goals are prioritized (Higgins et al., 1997). Goals perceived as necessities tend to command immediate attention and are less likely to be suppressed than those driven by aspirations or ideals (Freitas et al., 2002). Third, the relationship between focal and alternative goals is critical; when alternative goals are perceived as complementary to the primary goal, they may be integrated rather than suppressed (Shah et al., 2002). Individual differences also play an important role in goal-shielding processes. People with a stronger “need for closure” typically exhibit more pronounced goal-shielding tendencies as they prefer clear and unambiguous paths to goal attainment (Webster and Kruglanski, 1994). Similarly, emotional states like anxiety can enhance goal-shielding effects, particularly when the focal goal is viewed as a means of alleviating negative emotions (Shah et al., 2002).

In organizational contexts, goal-shielding theory has significant implications for understanding employees' behavior. Greenbaum et al. (2020) conceptualized bottom-line mentality (BLM) as a manifestation of goal shielding, wherein employees focus exclusively on bottom-line outcomes while neglecting other considerations, including ethical ones. This theoretical lens is particularly insightful because genuine organizational performance is not a monolithic target, but a complex construct determined by multiple, often competing financial criteria that require sophisticated evaluation (Kaya et al., 2024). Thus, BLM represents a cognitive narrowing onto select performance indicators at the direct expense of this broader, multifaceted reality. This helps explain why the different dimensions of employability may have such distinct impacts on an employee's propensity to engage in UPB (Kruglanski et al., 2018).

By understanding the goal-shielding mechanisms that underlie employees' responses to different employability conditions, we can better predict when and why employees might engage in unethical pro-organizational behavior. This theoretical foundation informs our subsequent hypotheses regarding the differential effects of internal and external employability on employees' ethical decision-making processes.

### 2.2 Internal employability and unethical pro-organizational behavior

Employees' internal employability represents their perception of the value they offer within their current organization, encompassing skills, knowledge, experience, and other attributes that are critical for sustaining or advancing one's career in a particular workplace (Rothwell and Arnold, 2007). Those with high internal employability believe that they possess the necessary competencies to maintain employment or advance within their current organization and feel secure in their position within it. Conversely, employees with low internal employability may perceive that they lack the skills needed to remain employed or advance at their current organization, leading them to experience a sense of job insecurity (Spurk et al., 2016). Drawing from goal-shielding theory, we argue that internal employability can influence UPB through BLM.

When employees perceive their internal employment capability is low, they are more likely to experience strong fear of losing their job. To avoid such a risk, they may prioritize keeping the current job or advancing within the organization as top goal. According to goal-shielding theory, when individuals are strongly committed to achieving one primary goal, they tend to suppress other goals that might interfere with their main focus (Shah et al., 2002). In this context, employees who perceive low internal employability may develop a BLM (Greenbaum et al., 2020). That is, prioritizing achieving performance goals over other goals and ethical considerations that might get in the way of progress of their main goal (Mawritz et al., 2024).

Furthermore, goal shielding theory suggests that the type of commitment can influence this process. Goals considered necessary or obligatory are likely to attract immediate attention and be prioritized over goals driven by aspirations or ideals (Higgins et al., 1997; Shah et al., 2002). For employees who perceive low internal employability, maintaining their job and advancing within the organization may represent a necessity rather than an aspiration (Callanan et al., 2017). This prevention-focused commitment can strengthen the goal shielding process, leading to greater prioritization of bottom-line results over competing goals such as moral considerations.

Moreover, the relationship between focal and alternative goals plays a crucial role in goal shielding. When an alternative goal is perceived as incompatible or even counterproductive to reaching the primary goal, it becomes more likely that this goal will be suppressed (Shah et al., 2002). Employees who perceive low internal employability may view adherence to ethical standards as a barrier to job stability and promotion, making them prioritize bottom-line results over moral behavior.

In contrast, employees with high internal employment capability are likely to feel more secure in their position within the organization. They may believe that they possess valuable skills and knowledge needed for both their job stability and career advancement. Consequently, these employees are less likely to develop a BLM because they do not feel the same pressure as those with low internal employability to prioritize bottom-line results over moral considerations in order to achieve long-term personal goals (Greenbaum et al., 2020; Mawritz et al., 2024). They may also perceive that adherence to ethical standards is compatible with their long-term career objectives within the organization, reducing the need for suppressing alternative goals such as moral behavior in order to focus on one performance goal. Based on the above reasoning, we propose the following hypotheses:

Hypothesis 1a: Employee's internal employability is negatively related to UPB.
Hypothesis 1b: Employee's bottom-line mentality mediates the negative relationship between internal employability and UPB.

### 2.3 External employability and UPB

Employee's external employment capability represents their perception of the value they offer outside their current organization, encompassing skills, knowledge, experience, and other attributes that are valuable in the external labor market (Rothwell and Arnold, 2007). According to goal-shielding theory, we propose a more complex relationship between external employability and UPB than internal employability, one that follows an inverted U-shaped pattern.

When employees perceive their external employment capability is low, they may feel “stuck” in the current job because of the limited job opportunities in the labor market. Unlike those with low internal employability, however, this lack of perceived external employability does not necessarily threaten one's current employment status (Guilbert et al., 2016; Rothwell and Arnold, 2007). Instead, it can motivate employees to align their long-term personal interests more closely with the success of the organization they are in. Therefore, those who perceive low external employability may be less likely to develop a BLM because they view their own long-term success as being tied to the moral reputation and overall success of the current organization rather than just short-term bottom-line results.

As external employability increases to a moderate level, employees may perceive that they possess some valuable skills and experience that are transferable to other employers but still feel uncertain about their ability to secure employment elsewhere when needed. This “middle” stage of uncertainty can motivate employees to prioritize demonstrating their value to potential future employers as a primary goal (Shah et al., 2002). To achieve this objective, they may focus on short-term performance metrics while suppressing other goals that might hinder their progress (Mawritz et al., 2024). As such, those with moderate external employability may be more likely to develop BLM, leading them to engage in UPB.

However, when employees perceive their external employment capability as high, they believe they possess highly transferable and valuable skills that are sought after by other employers. This perception provides a strong sense of cross-organizational career security because these employees feel confident in their ability to find alternative employment when needed (Guilbert et al., 2016). According to goal shielding theory, this confidence can reduce the need for employees to prioritize short-term performance objectives over moral considerations. Employees with high external employability may see adherence to ethical standards as compatible with maintaining a positive reputation in the external labor market and thus their long-term career success. Therefore, they are less likely to develop BLM because they do not feel compelled to suppress moral considerations in favor of short-term performance goals (Greenbaum et al., 2020). Thus, we propose:

Hypothesis 2a: There is a curvilinear (inverted U-shaped) relationship between employee's external employability and UPB, such that employees with moderate level external employability have greater possibility to engage in UPB compared to lower and higher levels of external employability.
Hypothesis 2b: Employee's bottom-line mentality mediates the curvilinear effect of external employability on UPB.

### 2.4 The moderating role of risk propensity

Risk propensity, defined as an individual's tendency to take risk (Nicholson et al., 2005), has been shown to vary significantly among individuals (Meertens and Lion, 2008; Zhang et al., 2019). We propose that this individual difference plays a crucial moderating role in the relationships between internal and external employability and UPB, particularly in the mediating role of BLM. As mentioned earlier, when individuals are strongly committed to a primary goal, they tend to suppress alternative goals that may interfere with their primary focus. However, the strength of this goal-shielding process can be influenced by individual differences (Shah et al., 2002), such as risk propensity. Employees with high risk propensity are more comfortable with uncertainty (Sitkin and Pablo, 1992), whereas those with low risk propensity tend to avoid risks and prioritize stability and security (Qu et al., 2025).

When employees perceive their internal employability to be low, they may experience job insecurity and prioritize maintaining their current position as their primary goal. For those with low risk propensity, this goal may become even more salient, as they are less comfortable with the uncertainty associated with potential job loss. As a result, they may be more likely to develop a BLM, focusing solely on bottom-line results and suppressing ethical considerations that could jeopardize their job security. In contrast, employees with high risk propensity may be more open to exploring alternative solutions to enhance their internal employability, such as acquiring new skills or seeking mentorship. They may view adherence to ethical standards as a calculated risk that could yield long-term benefits, such as positive reputation and increased trust within the organization. Consequently, the negative relationship between internal employability and UPB, mediated by BLM, may be stronger for employees with low risk propensity and weaker for those with high risk propensity.

Regarding external employability, employees with high risk propensity may be more comfortable with the uncertainty of the job market and more proactive in exploring external opportunities. Goal-shielding theory also proclaims that individuals with less need for closure and negative emotion states such as anxiety and depression will exhibit weaker goal-shielding process (Shah et al., 2002; Raufelder and Ringeisen, 2016). As a result, they may be less likely to develop a BLM and engage in UPB, even at moderate levels of external employability. On the other hand, employees with low risk propensity may be more sensitive to the uncertainty associated with moderate levels of external employability. They may perceive a greater need to prioritize short-term performance to secure their potential position in the external job market, leading to a stronger BLM and a higher likelihood of engaging in UPB. Furthermore, the relationship between focal and alternative goals may also be influenced by risk propensity. For employees with high risk propensity, adherence to ethical standards may be seen as more compatible with their long-term career goals, as they can be more resilient to short-term risks, therefore are willing to take calculated risks to maintain a positive reputation across organization. In contrast, those with low risk propensity may perceive ethical considerations as a more significant threat to their primary goal of maintaining employment, leading to a stronger tendency to suppress moral considerations in favor of bottom-line results. Based on these arguments, we propose the following hypotheses:

Hypothesis 3a: Employee's risk propensity moderates the relationship between internal employability and UPB, such that the negative effect is stronger under low risk propensity and attenuated under high risk propensity.
Hypothesis 3b: Employee's risk propensity moderates the curvilinear effect of external employability on UPB, such that the curvilinear effect is enhanced under low risk propensity and attenuated under high risk propensity.

## 3 Study 1

### 3.1 Sample and procedure

To test our hypotheses initially, we conducted a two-wave survey study within a large infrastructure company in southeastern China. A total of 300 employees were invited to participate. At Time 1, participants were asked to complete measures of external and internal employability, along with providing demographic information. All 300 employees invited returned completed questionnaires at Time 1. Two weeks later (Time 2), these employees were asked to complete a measure of unethical pro-organizational behavior; 273 questionnaires were returned, resulting in a final response rate of 91%. Of the 273 respondents, 69.2% were female, 80.2% were between 25 and 40 years old, 90.5% held at least a Bachelor's degree, and the distribution of organizational tenure was as follows: 1–3 years (24.5%), 4–6 years (31.1%), and 7–10 years (24.5%).

### 3.2 Measurements

Since our participants were Chinese and the original scales were in English, we followed the translation and back-translation procedures to translate all scales into Chinese versions (Brislin, 1970). Unless otherwise specified, a 5-point Likert scale was used for all measures, ranging from 1 = “strongly disagree” to 5 for “strongly agree.”

#### 3.2.1 Employability

In line with previous studies (Anasori et al., 2021; Koen et al., 2012), we assessed employability through the 11-item scale developed by Rothwell and Arnold (2007). The scale identified employability as one with two related components—external and internal employability. For the 7 items that measured external employability, a sample item is “The skills I have gained in my present job are transferable to other occupations outside this organization” (Cronbach α = 0.85). Regarding internal employability, of the original 4 items, we removed one to improve the scale's reliability. The remaining items are “Even if there was downsizing in this organization, I am confident that I would be retained”; “I am aware of the opportunities arising in this organization even if they are different to what I do now”; “Among the people who do the same job as me, I am well respected in this organization” (Cronbach α = 0.77).

#### 3.2.2 Unethical pro-organizational behavior

UPB was assessed by Umphress et al.'s (2010) six-item measure. A sample item is “If it would help my organization, I would misrepresent the truth to make my organization look good” (Cronbach α = 0.90).

#### 3.2.3 Control variables

We controlled employees' age, gender, education, and organizational tenure in our research, as prior studies indicated that demographic characteristics may affect employees' attitudes and behavior (Riordan and Shore, 1997; Li et al., 2008).

### 3.3 Results

Table 1 illustrates the means, standard deviations, and correlations among our focal variables. We conducted factor analysis to determine whether our measured variables are distinguishable from each other. The three-factor model, including external employability, internal employability, and unethical pro-organizational behavior, provides an acceptable fit (χ^2^ = 216.16, *df* = 116, CFI = 0.95, TLI = 0.95, RMSEA = 0.06, SRMR = 0.04) compared with one factor baseline model (χ^2^ = 966.88, *df* = 119, CFI = 0.61, TLI = 0.55, RMSEA = 0.16, SRMR = 0.14). Such evidence demonstrated discriminant and convergent validity.

**Table 1:** Study 1 means, standard deviations, and correlations of the variables.

**Variables**	***Mean***	***S.D*.**	**(1)**	**(2)**	**(3)**	**(4)**	**(5)**	**(6)**	**(7)**
AGE	2.49	0.89							
GEN	0.31	0.46	0.02						
EDU	4.09	0.63	−0.04	−0.15^*^					
Tenure	2.99	1.29	0.75^***^	0.04	−0.12^*^				
EEbility	3.83	0.68	0.12^*^	0.05	0.19^**^	0.18^**^	(0.85)		
IEbility	3.89	0.64	0.21^***^	0.03	0.17^**^	0.29^***^	0.64^***^	(0.77)	
UPB	3.24	1.31	−0.26^***^	0.03	−0.11	−0.27^***^	−0.30^***^	−0.46^***^	(0.90)

*N* = 273. Values in the brackets are Cronbach's alpha coefficients. ^*^p < 0.05, ^**^p < 0.01, ^***^p < 0.001.

As shown in Table 2, the regression analysis' results demonstrate that both hypothesis 1a and hypothesis 2a were supported by the data. Specifically, internal employability is negatively related to UPB (Model 2; B = −0.52, SE = 0.09, *p* < 0.001), while external employability is not significantly related to UPB (Model 2; B = −0.02, SE = 0.09, *p* > 0.05). Furthermore, the quadratic term of external employability is negatively related to UPB (Model 3; B = −0.26, SE = 0.12, *p* < 0.05), suggests there is an inverted U-shaped relationship between external employability and UPB.

**Table 2:** Study 1 results of hierarchical regression analysis.

**Variables**	**UPB**		
**Model 1**	**Model 2**	**Model 3**
*Intercept*	5.38^***^	4.23^***^	4.30^***^
**Control variables**			
AGE	−0.19 (0.13)	−0.22 (0.12)	−0.25^*^ (0.12)
GEN	0.04 (0.17)	0.11 (0.15)	0.12 (0.15)
EDU	−0.27 ^**^ (0.12)	−0.09 (0.16)	−0.07 (0.11)
TENURE	−0.19^*^ (0.09)	−0.04 (0.08)	−0.02 (0.08)
**Independent variables**			
IEbility		−0.52^***^ (0.09)	−0.50^***^ (0.09)
EEbility		−0.02 (0.09)	−0.16 (0.11)
EEbility^2^			−0.26^*^ (0.12)
*R^2^*	0.10	0.24	0.26

*N* = 273. Values in the brackets are standard errors.

^*^p < 0.05, ^**^p < 0.01, ^***^p < 0.001.

## 4 Study 2

### 4.1 Sample and procedure

In Study 2, we collected data from Credamo, which is one of the largest Chinese online survey platforms providing sample service (Xing et al., 2024; Zhang et al., 2022). We used a pre-set filter to conduct the survey that is exclusively targeted at employees who are currently employed. To make sure our participants are real genuine employees from profit-oriented companies throughout China, we employed several additional measures provided by the platform, including (1) participants are required to complete identity verification before answering the questionnaire minimize bot answer. (2) We restricted participation to those who had completed fewer than 10 surveys on Credamo to exclude potential professional questionnaire fillers. (3) Participants from each IP address can answer once for one wave survey to lower the possibility that one respondent uses multiple accounts answering the same questionnaire. (4) Two participants' geographic location >10 km were required to further maximize the independence of each survey response. We collected data in three different times, separated by 2 weeks. At Time 1, participants provide information about their internal and external employability, risk propensity, and their demographic characteristics such as age, gender, education, tenure, and organization type. Four hundred fifty questionnaires were collected at this time. After reviewing the data, we excluded three participants who reported their organization type is others and stopped distributing subsequent questionnaires to them. At Time 2, two weeks later, we distributed the questionnaires to these 450 participants asking them to rate on their BLM and obtained 358 questionnaires. At time 3, participants were asked to rate on their UPB, and 316 participants responded.

Among the participants, the average age was 31.59 years (SD = 5.60), 34.8% were male, 67.1% has a bachelor's degree and 26.3% has a master's degree or above, and their average organizational tenure was 7.4 years (SD = 4.18). In addition, for the organization type they are currently worked in, 57.6% was from private domestic company, 28.2% was from state-owned company, and 14.2% was from foreign-invested company.

### 4.2 Measurements

Since our participants were Chinese and the original scales were in English, we followed the translation and back-translation procedures to translate all scales into Chinese versions (Brislin, 1970). Unless otherwise specified, a 5-point Likert scale was used for all measures, ranging from 1 = “strongly disagree” to 5 for “strongly agree.”

#### 4.2.1 Employability

As in Study 1, Rothwell and Arnold's (2007) 11 item scale was used to measure employee's internal and external employability (Cronbach α = 0.65).

#### 4.2.2 Unethical pro-organizational behavior

As in Study 1, Umphress et al.'s (2010) 6 item scale was used to measure employee's unethical pro-organizational behavior (Cronbach α = 0.87).

#### 4.2.3 Bottom-line mentality

We measured BLM by adopting a 4-item scale developed by Greenbaum et al. (2012). A sample item is “I only care about the business” (Cronbach α = 0.89).

#### 4.2.4 Risk propensity

A 7-item scale developed by Meertens and Lion (2008) was used to measure employee's risk propensity. In order to measure respondents' risk-seeking tendency, four items were reverse-scored. Higher scores on the RPS indicate higher risk-seeking tendencies. All statements were rated on a 5-point scale ranging from 1 (totally disagree) to 5 (totally agree), except for the last item, which was rated on a scale ranging from 1 (risk avoider) to 5 (risk seeker). A sample reverse-scored item is “I do not take risks with my health” (Cronbach α = 0.89).

#### 4.2.5 Control variables

We controlled several variables that could influence UPB or that might offer alternative explanations for our findings. First, given that demographic factors could be an important source relevant for UPB, following convention, we controlled employee's age, gender, education, and tenure. Second, previous studies demonstrated that organizational culture and employee's attitudes differ in company's ownership structure (Tsui et al., 2006). Because our participants come from different types of organizations (i.e., private company, state-owned company, and foreign company), we therefore recoded organization type into dummy variable and included it as control. Third, our participants come from 25 out of 34 different provincial administrative regions across China, each with varying levels of economic development. It stands to reason that regions with more developed economies might boast more robust labor markets and a higher concentration of company headquarters compared to their less developed counterparts. Therefore, to account for regional economic disparities' influence on our hypothesized relationships, we included the GDP per capita figures for the year 2023 for each region, as reported by the Chinese Bureau of Statistics, as an additional control variable in our analysis.

### 4.3 Results

Table 3 presents the means, standard deviations, and correlations among the study variables. We first conducted a confirmatory factor analysis using lavaan package in R language to determine whether our measured variables are distinguishable from each other. We examined the five-factor model that included internal employability, external employability, UPB, BLM, and risk propensity. This model exhibited acceptable fit to the data (χ^2^ = 759.72, *df* = 314, CFI = 0.91, TLI = 0.90, RMSEA = 0.07, SRMR = 0.07). We further tested the discriminant validity of the five constructs by comparing the fit of the hypothesized five-factor model with alternative models. All the alternative models provided poor fits to the data (see Table 4).

**Table 3:** Study 2 means, standard deviations, and correlations of the variables.

**Variables**	***Mean***	***S.D*.**	**(1)**	**(2)**	**(3)**	**(4)**	**(5)**	**(6)**	**(7)**	**(8)**	**(9)**	**(10)**	**(11)**	**(12)**
AGE	31.59	5.6												
GEN	0.35	0.48	0.13^*^											
EDU	2.2	0.54	0.08	0.01										
Tenure	7.4	4.18	0.90^***^	0.15^**^	−0.04									
Gdpp	9.87	3.62	0.15^**^	−0.01	0.07	0.12^*^								
Otype1	0.58	0.49	0.09	−0.13^*^	−0.03	0.02	0.01							
Otype2	0.14	0.35	−0.07	0.06	−0.08	−0.04	0.05	−0.47^***^						
EEbility	4.12	0.62	0.12^*^	0.13^*^	0.21^***^	0.07	0.11	−0.04	−0.15^**^	(0.85)				
IEbility	4.22	0.52	0.25^***^	0.16^**^	0.21^***^	0.21^***^	0.08	−0.09	−0.06	0.62^***^	(0.65)			
BLM	2.16	0.97	−0.12^*^	−0.02	−0.34^***^	−0.02	−0.15^**^	0.01	0.1	−0.32^***^	−0.51^***^	(0.89)		
UPB	2.09	0.84	−0.19^***^	0.02	−0.23^***^	−0.1	−0.14^**^	−0.01	0.12^*^	−0.26^***^	−0.34^***^	0.63^***^	(0.87)	
RP	3.07	0.98	−0.05	−0.06	0.20^***^	−0.08	0.13^*^	−0.02	−0.08	0.29^***^	0.35^***^	−0.46^***^	−0.38^***^	(0.89)

*N* = 316. Values in the brackets are Cronbach's alpha coefficients.

Gender was dummy coded as 0 (= male) and 1 (= female). Education was coded as a three-level categorical variable: 1 = below bachelor, 2 = bachelor, 3 = above bachelor. Gdpp is province level GDP per capita in 2023. Otype1 and Otype2 are dummy variables created from organization type. EEbility, external employability; IEbility, internal employability; BLM, bottom-line mentality; UPB, unethical pro-organizational behavior; RP, risk propensity.

^*^p < 0.05, ^**^p < 0.01, ^***^p < 0.001.

**Table 4:** Study 2 comparison of measurement models.

**Model**	***χ^2^***	***df***	***χ^2^*/*df***	**CFI**	**TLI**	**RMSEA**	**SRMR**
EEbility, IEbility, RP, BLM, UPB	759.72	314	2.42	0.91	0.90	0.07	0.07
EEbility + IEbility, RP, BLM, UPB	818.11	318	2.57	0.89	0.89	0.07	0.08
EEbility + IEbility + BLM, RP, UPB	1,576.84	321	4.91	0.74	0.71	0.11	0.13
EEbility + IEbility + RP, BLM, UPB	1,792.29	321	5.58	0.69	0.67	0.12	0.13
EEbility + IEbility + BLM + RP, UPB	2,501.72	323	7.75	0.55	0.51	0.15	0.14
EEbility + IEbility + RP + BLM + UPB	2,799.49	324	8.64	0.48	0.44	0.16	0.14

“+” indicates factors combined. EEbility, external employability; IEbility, internal employability; BLM, bottom-line mentality; UPB, unethical pro-organizational behavior; RP, risk propensity.

Hypothesis 1a posits that internal employability is negatively related to employee's UPB, As shown in Model 8 in Table 5, the coefficient of internal employability on UPB is −0.32 (SE = 0.11, *p* < 0.01). Supporting Hypothesis 2a, external employability is not significantly related to UPB (B = −0.05, SE = 0.09, *p* > 0.05) in Model 7, but the quadratic term of external employability is negatively related to UPB (Model 8; B = −0.34, SE = 0.10, *p* < 0.01).

**Table 5:** Results of hierarchical regression analysis.

**Variables**	**BLM**					**UPB**			
	**Model 1**	**Model 2**	**Model 3**	**Model 4**	**Model 5**	**Model 6**	**Model 7**	**Model 8**	**Model 9**
*Intercept*	4.94^***^ (0.51)	4.09^***^	4.05^***^	4.07^***^	4.89^***^	4.36^***^	3.89^***^	3.84^***^	1.82^***^
**Control variables**									
AGE	−0.07^**^ (0.02)	−0.05^*^ (0.02)	−0.04^*^ (0.02)	−0.05^**^ (0.02)	−0.05^**^ (0.02)	−0.06^**^ (0.02)	−0.05^**^ (0.02)	−0.05^*^ (0.02)	−0.03 (0.02)
GEN	−0.04 (0.11)	0.07 (0.10)	0.09 (0.10)	−0.00 (0.09)	0.04 (0.09)	0.05 (0.09)	0.12 (0.09)	0.14 (0.09)	0.10 (0.08)
EDU	−0.49^***^ (0.10)	−0.35^***^ (0.09)	−0.34^***^ (0.09)	−0.29^***^ (0.08)	−0.29^***^ (0.08)	−0.26^**^ (0.09)	−0.18^*^ (0.09)	−0.18^*^ (0.09)	−0.01 (0.07)
Tenure	0.08^**^ (0.03)	0.08^**^ (0.03)	0.07^**^ (0.03)	0.07^**^ (0.02)	0.07^**^ (0.02)	0.06^*^ (0.03)	0.06^*^ (0.03)	0.05 (0.03)	0.01 (0.02)
Gdpp	−0.03^*^ (0.01)	−0.03^*^ (0.01)	−0.03^*^ (0.01)	−0.03^*^ (0.01)	−0.02 (0.01)	−0.03^*^ (0.01)	−0.02 (0.01)	−0.02 (0.01)	−0.01 (0.10)
Otype1	0.17 (0.12)	0.04 (0.11)	0.01 (0.11)	0.01 (0.10)	0.02 (0.10)	0.14 (0.11)	0.07 (0.10)	0.03 (0.10)	0.03 (0.09)
Otype2	0.32 (0.16)	0.19 (0.15)	0.17 (0.15)	0.18 (0.14)	0.13 (0.14)	0.31^*^ (0.15)	0.23 (0.14)	0.20 (0.14)	0.12 (0.12)
**Independent variables**									
IEbility		−0.91^***^ (0.11)	−0.82^***^ (0.12)	−0.27 (0.14)	−0.61^***^ (0.12)		−0.42^***^ (0.11)	−0.32^**^ (0.11)	0.09 (0.10)
EEbility		0.07 (0.09)	−0.28 (0.16)	−0.42^**^ (0.15)	−0.28 (0.15)		−0.05 (0.09)	−0.46^**^ (0.15)	−0.32^*^ (0.13)
EEbility^2^			−0.29^**^ (0.11)	−0.45^***^ (0.10)	−0.36^**^ (0.11)			−0.34^**^ (0.10)	−0.19^*^(0.09)
RP				−0.34^***^ (0.05)	−0.25^***^ (0.05)				
BLM									0.50^***^ (0.05)
**Interaction variables**									
IEbility × RP				0.47^***^ (0.11)					
EEbility^2^ × RP					−0.09 (0.07)				
*R^2^*	0.16	0.35	0.36	0.45	0.43	0.12	0.19	0.22	0.43

*N* = 316. Values in the brackets are standard errors.

^*^p < 0.05, ^**^p < 0.01, ^***^p < 0.001.

The proposed indirect and conditional indirect effects were examined using bootstrapping-based approach proposed by Edwards and Lambert (2007). Unlike traditional approaches to test the significance of indirect effects that assume a normal sampling distribution of the product term of two regression coefficients, bootstrapping is a nonparametric statistical technique that does not require an a priori assumption about the shape of the sampling distribution for this product term (Efron, 1979). This approach has been used to test indirect effects in mediated models (MacKinnon et al., 2004; Shrout and Bolger, 2002) and can be extended to models that combine mediation and moderation as well (Edwards and Lambert, 2007). Following these guidelines, we set the resample times to 10,000.

**Figure 2:**
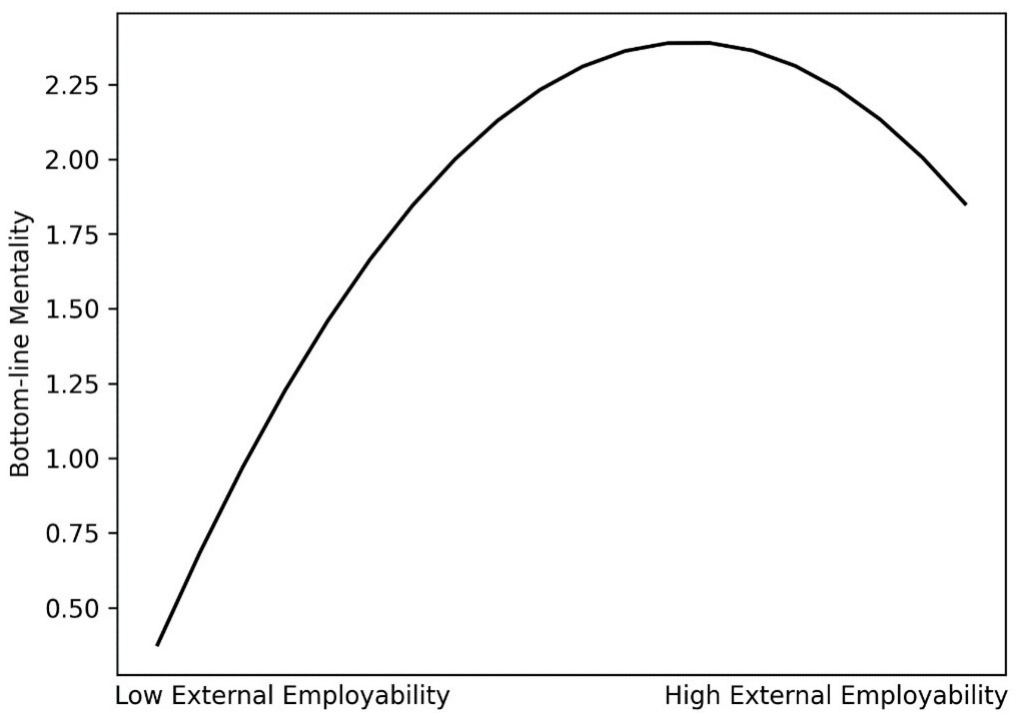
The curvilinear relationship between external employability and bottom-line mentality.

In support of Hypothesis 1b, the negative indirect effect of internal employability on UPB was mediated by BLM [indirect effect = −0.47, SE = 0.08, 95% CI (−0.64, −0.32)]. Regarding Hypothesis 2b, we followed Hayes and Preacher's (2010) recommendations to examine the suggested curvilinear indirect relation. Hypothesis 2b suggested an indirect curvilinear relationship between external employability and UPB via BLM. The squared term of external employability was negatively related to BLM (B = −0.29, SE = 0.11, *p* < 0.01; Table 5, Model 3). This curvilinear, inverted U-shaped relation is depicted in Figure 2. Furthermore, BLM is positively related to UPB (B = 0.50, SE = 0.05, *p* < 0.001; Table 5, Model 9).

Overall, this pattern suggests the possibility of an indirect curvilinear relationship between external employability and UPB through BLM. To further test this notion, we calculated the instantaneous indirect effects of external employability on UPB through BLM at different external employability values (i.e., the means as well as 2 SD and 1 SD around the mean; cf. Hayes and Preacher, 2010). Corroborating the anticipated inverted U-shape, these instantaneous indirect effects were positive at −2 SD [estimate = 0.22, SE = 0.10; 95% CI = (0.04, 0.41)] and −1 SD [estimate = 0.04, SE = 0.05; 95% CI = (−0.05, 0.13)], nonsignificant at the mean [estimate = −0.14, SE = 0.10; 95% CI = (−0.33, 0.05)], and negative at +1 SD [estimate = −0.32, SE = 0.17; 90% CI = (−0.60, −0.02)] and +2 SD [estimate = −0.50, SE = 0.23; 90% CI = (−0.91, −0.07)]. Therefore, Hypothesis 2b was supported.

**Figure 3:**
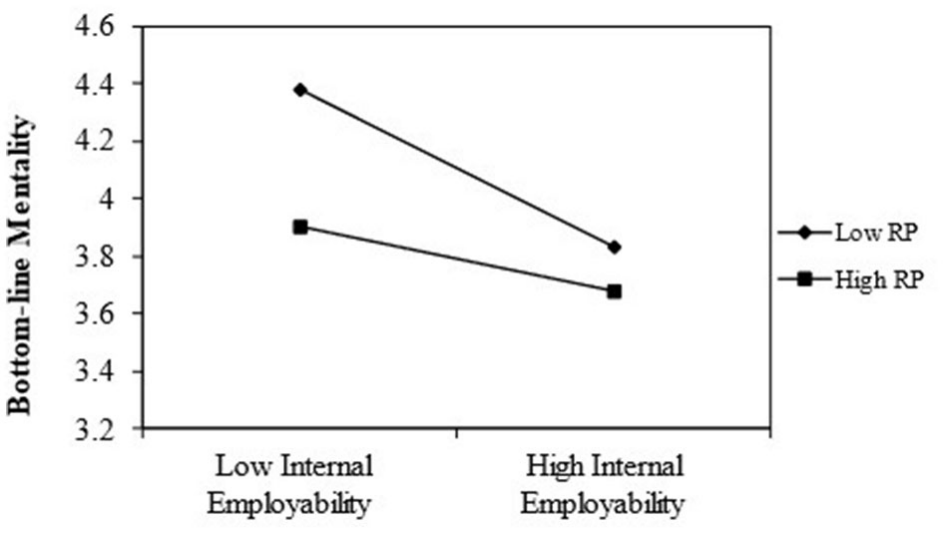
Interactive relationship of internal employability and risk propensity with BLM.

Hypothesis 3a proposed a moderating role of risk propensity for the indirect relationship between internal employability and UPB through BLM. As shown in Table 5, the interaction coefficient for internal employability and risk propensity was significantly related to BLM (B = 0.47, SE = 0.11, *p* < 0.001; Table 5, Model 4). In other words, risk propensity buffered the negative relationship between internal employability and BLM. Figure 3 illustrates the pattern of this interaction. Moreover, when risk propensity was low (−1SD), the indirect relationship was negative [estimate = −0.43, SE = 0.07; 95% CI = (−0.58, −0.29)]. When risk propensity was higher, by contrast, the indirect relationship was nonsignificant [estimate = −0.12, SE = 0.10; 95% CI = (−0.35, 0.07)]. In addition, there was a significant difference between the two conditional indirect relationships [estimate = 0.31, SE = 0.10; 95% CI = (0.11, 0.51)]. Therefore, hypothesis 3a was supported.

Hypothesis 3b suggested that risk propensity moderates the indirect curvilinear relationship between external employability and UPB through BLM. As shown in Table 5, however, the interaction coefficient between the squared term of external employability and risk propensity was not significantly related to BLM (B = −0.09, SE = 0.07, *p* > 0.05; Table 5, Model 5). Hence, it's clear that risk propensity did not have the suggested moderating role in the indirect linkage from external employability to UPB via BLM, refuting hypothesis 3b.

## 5 Discussion

Our research set out to unravel the complex and often contradictory ways in which an employee's career prospects—both inside and outside their organization—shape their ethical behavior. The findings from our two studies paint a nuanced psychological picture. On one hand, feeling secure and valued internally acts as a protective shield, liberating employees from a narrow survival-focused mindset and thereby reducing their propensity for unethical pro-organizational behavior (UPB). On the other hand, the journey of building external employability presents a more perilous path, with a moderate level of perceived opportunity paradoxically increasing the risk of UPB. These differential effects, mediated by a bottom-line mentality (BLM), are further qualified by an employee's risk propensity, though in a more complex manner than anticipated. These findings not only make significant contributions to the literature on careers and ethics but also carry critical implications for managerial practice.

### 5.1 Theoretical contribution

The present research makes several significant theoretical contributions to the literature on career development and UPB. First, we introduce employability as a new antecedent of UPB, providing a nuanced understanding of how its two dimensions, internal and external employability, distinctively influence employees' likelihood of engaging in UPB. While prior research has explored various antecedents of UPB, such as personality traits (Castille et al., 2018), organizational culture (Vem et al., 2023), and leadership styles (Bryant and Merritt, 2021), the role of career-related factors, particularly employability, has been largely overlooked. Our findings bridge this gap and extend the current understanding of the drivers of UPB by demonstrating those employees' perceptions of their internal and external employability can significantly impact their ethical decision-making processes.

Moreover, this research contributes to the literature on employability and career research by revealing the important implications of employability for employees' ethical decision-making, beyond its well-documented consequences for career transitions (De Vos et al., 2021; Farashah et al., 2023) and employee retention (Acikgoz et al., 2016; Nelissen et al., 2017). Our findings highlight the need for a more comprehensive understanding of the consequences of employability, not only in terms of traditional career outcomes but also in terms of employees' ethical decision-making processes. This expanded perspective can inform future research on the far-reaching effects of employability on various aspects of employee behavior that are related to business ethics.

Furthermore, we reveal the mediating role of bottom-line mentality (BLM) in these relationships, offering a clear psychological mechanism. Drawing explicitly from goal-shielding theory (Shah et al., 2002), our findings show that low internal employability triggers a powerful focal goal: “protect my job at all costs.” This intense focus effectively shields out competing, non-instrumental goals, such as adhering to societal ethics, resulting in an amplified BLM and, consequently, a higher likelihood of UPB. In essence, it cognitively narrows an employee's world to the single-minded pursuit of performance outcomes. By empirically demonstrating this pathway, we extend the application of goal-shielding theory into the career-ethics domain and specify how internal job security translates into ethical conduct.

In contrast, we find an inverted U-shaped relationship between external employability and UPB via BLM. This non-linear effect uncovers the unique psychological precarity of employees at a moderate level of external employability—those who perceive themselves as “good, but not good enough.” This state of heightened career uncertainty appears to be a powerful catalyst for a myopic focus on short-term performance signaling. Employees at this stage are not merely insecure; they are in an active, high-stakes campaign to prove their value to a potential external market. This may trigger a goal-shielding process where the goal of “enhancing my external resume” overrides ethical considerations, leading to higher BLM. Interestingly, at both low levels (where employees feel “stuck” and align with their firm's long-term reputation) and high levels (where employees feel secure and protect their personal reputation), this pressure is attenuated, highlighting the unique risks of being in career transition limbo.

Additionally, we enrich this model by identifying employee risk propensity as a key boundary condition. Specifically, the negative indirect effect of internal employability on UPB (via BLM) is strongest for employees with low risk propensity. This finding suggests that the threat of low internal employability is perceived most acutely by those who are naturally risk-averse. For these individuals, the insecurity triggers a maximal goal-shielding response—a desperate focus on the bottom line to restore a sense of security. Conversely, employees with high risk propensity appear more resilient; they may view low internal employability less as a terminal threat and more as a challenge to be navigated, thus weakening the link between their job insecurity and a bottom-line obsession. This offers a more contextualized understanding of who is most vulnerable to the ethical risks of job insecurity.

Perhaps our most theoretically instructive finding is the lack of support for the hypothesized moderating role of risk propensity in the relationship between external employability and UPB (Hypothesis 3b). This null finding is instructive when contrasted with the significant moderation found for internal employability. It suggests that the psychological pressures associated with external employability, particularly the career uncertainty at moderate levels, may constitute a “strong situation” that tends to override an individual's dispositional risk tolerance. While an employee's general risk propensity strongly colors their reaction to internal job threats, the specific challenge of navigating the external job market and managing one's professional reputation appears to be governed by a more specific calculus of reputational risk that is distinct from general risk-taking tendencies. This distinction underscores the unique nature of external employability and points to a promising avenue for future research to explore other potential moderators, such as career stage or social network strength, that might shape this complex relationship.

### 5.2 Practical contributions

Our research findings offer several practical implications for organizations and managers seeking to promote ethical behavior and prevent UPB among their employees. First, our results highlight the importance of fostering employees' internal employability as a means of reducing the likelihood of UPB. Organizations should invest in training and development programs that enhance employees' skills, knowledge, and competencies, thereby increasing their perceived value within the organization. By providing opportunities for career growth and advancement, managers can help employees feel more secure in their current positions and less likely to engage in UPB as a means of protecting their jobs. Second, our findings offer a crucial warning for managers: be particularly mindful of employees with moderate levels of external employability. While these individuals may appear ambitious and high-performing, their underlying career uncertainty makes them uniquely susceptible to engaging in UPB. Managers should not mistake their intense focus on results for unwavering organizational loyalty. Instead, they should engage these employees in targeted career development conversations, provide mentorship, and explicitly integrate ethical conduct into performance evaluations. Reinforcing that long-term success (both internal and external) is built on integrity can help counteract the short-term focus, bottom-line mentality that this career stage can foster. Third, our research underscores the significance of promoting a strong ethical culture within organizations. By emphasizing the importance of ethical behavior and making it a core value of the organization, managers can help employees prioritize ethical considerations over short-term performance goals. This can be achieved through regular ethics training, clear communication of ethical standards, and consistent enforcement of consequences for unethical behavior. By fostering a culture that values integrity and responsibility, organizations can reduce the risk of UPB and create a more positive work environment. Finally, our findings regarding the moderating role of risk propensity suggest that managers should consider individual differences when assessing the likelihood of UPB among their employees. Employees with low risk propensity may be more susceptible to the negative effects of low internal employability on UPB, while those with high risk propensity may be less influenced by these factors. Managers can use this information to tailor their approaches to promoting ethical behavior, providing additional support and guidance to employees who may be more vulnerable to engaging in UPB due to their risk-taking tendencies.

### 5.3 Limitations and future directions

While the present research makes significant contributions to the literature on employability and unethical pro-organizational behavior (UPB), it is not without limitations that offer opportunities for future research. First, though we conducted two studies to provide support for our theoretical model, our second study relied on self-reported measures because of practical limitations when using online survey platforms, which may be subject to common method bias. Nevertheless, we think it worth sacrificing a certain degree of methodology rigor in accessing a way more representative nationwide data which may greatly increase our research's external validity. This allows us to randomly survey employees across various organizations located in most of China's provinces instead of utilizing convenient sampling methodology limiting sample in one or a few organization(s) in one area. Moreover, the current research is related to employee's ethical considerations, recruiting participants from online survey platforms is more likely to earn respondents' trust to mitigate social desirability bias compared to traditional ways that requesting HR departments to distribute questionnaires within their own organization. Furthermore, we took steps to mitigate this concern, such as three waves of data collection. We also conducted a Harman one-factor test (Podsakoff et al., 2003), which indicated that CMV is unlikely to be a significant issue in our results. However, future research could employ alternative methods, such as peer or supervisor ratings, to assess these constructs and enhance the objectivity of the findings.

Second, while our research examined the distinct effects of internal and external employability on UPB, we didn't investigate how these two dimensions of employability interact to influence employees' ethical decision-making. Future studies could address on this issue by employing response surface methodology to delve into the different combinations of internal and external employability and their impacts on UPB. Exploring these interactions could provide a more comprehensive understanding of how employability shapes employees' ethical behavior in the workplace.

Third, our research was conducted in the context of Chinese organizations, which may limit the generalizability of our findings to other cultural contexts. Future research could replicate and extend our findings in diverse cultural settings to examine the cross-cultural validity of the proposed relationships. Such research could also explore how cultural values, such as collectivism or power distance, may moderate the effects of employability on UPB.

Fourth, we acknowledge a psychometric limitation concerning our measure of internal employability in Study 2. The Cronbach's alpha for this scale was 0.65, which is below the conventional 0.70 threshold for acceptable reliability (Nunnally, 1978). While values in this range are sometimes considered tolerable in exploratory research, particularly for scales with a small number of items (Hair et al., 2010), this inconsistency between our two studies warrants caution. Nevertheless, this lower reliability may be due to sample-specific characteristics or the nuances of the online data collection context. Therefore, we echo the need for future research to validate our findings using alternative and potentially more robust, measures of internal employability to ensure the stability of this important effect.

Finally, our research highlights the importance of considering employability as a key antecedent of UPB, opening new avenues for future research on the role of career-related factors in shaping employees' ethical decision-making. Future studies could explore how other career-related constructs, such as career adaptability, career commitment, or career stage, may influence employees' propensity to engage in UPB. Additionally, researchers could investigate how organizations' career development practices, such as mentoring, training, or performance appraisal systems, may shape employees' perceptions of their employability and subsequent ethical behavior.

## Data Availability

The raw data supporting the conclusions of this article will be made available by the authors, without undue reservation.
